# B cells characterization in ADA2 Deficiency patients

**DOI:** 10.1186/1546-0096-13-S1-P65

**Published:** 2015-09-28

**Authors:** F Schena, S Volpi, R Caorsi, C Pastorino, F Penco, F Kalli, A Omenetti, S Chiesa, A Bertoni, P Picco, G Filaci, I Aksentijevich, A Grossi, I Ceccherini, A Martini, E Traggiai, M Gattorno

**Affiliations:** 1 Gaslini Institute, Second Pediatric division, Genoa, Italy; 2 University of Genoa, CEBR, Genova, Italy; 3 National Institute of Health, National Human Genome Research Institute,, Bethesda, USA; 4 Gaslini Istitute, Medical Genetics, Genova, Italy; 5 Novartis Institute for Research in Biomedicine, Basel, Switzerland

## Introduction

ADA2 deficiency, a recently described disease, is characterized by systemic vasculopathy and episodes of strokes. The defect is due to a loss of function mutation of *CECR1* gene, codifying for Adenosine Deaminase 2 protein. This protein regulates the catabolism of extracellular adenosine, which we have recently shown is an important regulator of Class Switch Recombination in B lymphocytes. Accordingly DADA2 patients can present hypogammaglobulinemia.

## Objectives

Therefore we decided to characterize peripheral B and T lymphocytes of DADA patients to directly address if ADA2 mutation affects B-cell function and in particular we focused on B cell- T cell interaction.

## Patients and methods

3 patients carrying mutations in *CECR1* were examined. They showed clinical history with livedo reticularis, fever, vasculitis and neurological symptoms. Two patients presented hypogammaglobulinemia requiring intravenous immunoglobulin replacement therapy. We analyzed peripheral B and T cell phenotype by flow cytometry, *in vitro* B-cell proliferation and differentiation to Immunoglobulin secreting cells in response to CpG and T cell help.

## Results

Flow cytometer analysis showed a reduction of total B cells compared with age matched controls. Intriguingly a decrease in the percentage of memory B cell compartment (CD19+CD27+) was observed. Moreover we noted that the rate of B cells proliferation and differentiation to Immunoglobulin Secreting Cells of DADA2 patients with autologous T cell help are impaired. In fact in vitro IgM, IgG and IgA secretion is significantly reduced with respect to HD B lymphocytes in presence of mutated CD4 helper T cells.

## Conclusions

Our findings suggest that ADA2 defect could lead to a defect in B cell function and to a reduced T cell dependent B cell response.

